# Childhood adversity trajectories and weight status in young adult men: a register-based study including 359,783 Danish men

**DOI:** 10.1038/s41366-024-01540-4

**Published:** 2024-05-30

**Authors:** Cathrine L. Wimmelmann, Christoffer Sejling, Rebecca B. Clarke, Leonie K. Elsenburg, Thorkild I. A. Sørensen, Naja H. Rod

**Affiliations:** 1 Section of Environmental Health, Department of Public Health, University of Copenhagen, Øster Farimagsgade 5A, Copenhagen, Denmark; 2 Centre for Childhood Health, Islands Brygge 41, 2300 Copenhagen S, Copenhagen, Denmark; 3 Section of Biostatistics, Department of Public Health, University of Copenhagen, Øster Farimagsgade 5A, Copenhagen, Denmark; 4 Section of Epidemiology, Department of Public Health, University of Copenhagen, Øster Farimagsgade 5A, Copenhagen, Denmark; 5 Section on Genomic Physiology and Translation, Novo Nordisk Foundation Center for Basic Metabolic Research, University of Copenhagen, Blegdamsvej 3B, 2200 Copenhagen N, Copenhagen, Denmark

**Keywords:** Epidemiology, Risk factors

## Abstract

**Background:**

Childhood adversity has previously been associated with overweight and obesity in adult life, but there is a need for larger population-based studies using prospectively obtained adversity trajectories across childhood to confirm these associations. Moreover, childhood adversity may also be associated with underweight, which is less often studied. The aim of the current study is to investigate the association between childhood adversity trajectories from 0–15 years with weight categories in young adult men.

**Methods:**

The Danish Life Course Cohort (DANLIFE) was linked with the Danish Conscription Registry resulting in a study sample of 359,783 men, who have been assigned to one of five previously identified adversity trajectories from 0–15 years: “low adversity”, “early material deprivation”, “persistent material deprivation”, “loss or threat of loss”, and “high adversity”. Height and weight in young adulthood was assessed at a draft board examination at age 18–26 years. Associations of adversity trajectories and weight categories were investigated in multinomial regression models.

**Results:**

Compared with the “low adversity” group, the four other adversity groups had higher risks of underweight, overweight, and obesity. The “high adversity” group showed the strongest associations with both underweight (1.44 (1.32, 1.58)) and obesity (1.50 (1.39, 1.61)) when adjusted for parental origin, birth year, age at draft board examination, and maternal age.

**Conclusion:**

Childhood adversity, experienced between 0 and 15 years of life, was associated with a higher risk of underweight, overweight, and obesity in young adulthood among men.

## Introduction

A rapid and continued rise in overweight and obesity in most parts of the world is of major public health concern. Overweight and obesity have serious short and long-term consequences for physical, mental, and socioeconomic aspects of life [1]. Research has indicated that especially weight status in young adulthood (around ages 20–30) is of importance for later health outcomes [2, 3]. The path to young adult obesity may be established during childhood [4], making it central to study childhood predictors of body mass index (BMI) in young adulthood. Along these lines, adverse childhood experiences have received increasing attention in recent years as a potential risk factor for unhealthy weight development [5]. Childhood adversity can be defined as intense stressors or traumatic events that disrupt a child’s sense of safety and stability, potentially leading to mental and physiological consequences that can extend into adulthood [6]. Studies have shown that such childhood adversities are common, even in developed countries where reports indicate that around half of all adults have experienced at least one type of adversity [7–9].

Childhood adversities, such as parental divorce, poverty, parental or sibling death, and family drug abuse, have been found to have strong and enduring effects on health in adulthood [5, 6, 9, 10] and systematic reviews have consistently reported that individuals who have experienced adversities in childhood have higher risk of developing overweight and obesity in adulthood [11, 12]. Several potential mechanisms for the association between childhood adversity and later obesity have been proposed including both biological and stress-related pathways [13, 14], and behavioral pathways [15–17]. Furthermore, according to both the Food Insecurity Hypothesis [18, 19] and the more recent Adiposity Force Theory [20], the perception of poor social resources, which is a consequence of many types of adversities [21], triggers a mechanism encoding the body to store fat [20]. Thus, both theories propose the existence of an evolutionary developed biological reaction to difficult early life conditions, which increases the risk of developing overweight and obesity.

Though the association between childhood adversity and obesity in adulthood is relatively well-established [5], several questions remain unresolved. Previous studies are mainly based on selected and small samples, and there is a need for studies replicating the findings in large population-based samples. Also, the majority of the previous studies have used retrospectively obtained measures of single adversities [9, 22–25], but adversities tend to cluster and focusing on single events may have resulted in an underestimation of the effect of adversities on weight status. Studies investigating the accumulation of adverse events in childhood with weight status have generally found a dose-response relation between number of adversities and obesity [9, 26]. Finally, most studies used self-reported measures of BMI [22, 24, 25] and investigated associations between adverse experiences in childhood and obesity defined as BMI ≥ 30 kg/m^2^ rather than the full range of weight categories. However, it has been indicated that adversities in childhood may also be associated with underweight in adulthood, yet results are inconsistent [22].

The aim of the present study is to investigate associations of adversity trajectories (prospectively obtained from 0–15 years) on the whole range of weight categories in adulthood (underweight, normal weight, overweight, and obesity) in a large population-based sample of Danish men. Based on theoretical and empirical considerations, it was hypothesized that men who experienced adversities in childhood would show greater risk of both underweight, overweight, and obesity compared with those who have experienced no or few adversities during childhood.

## Methods

### Study sample

The Danish Life Course Cohort (DANLIFE) [27] was linked with the Danish Conscription Registry (DCR) [28] to investigate the aim of the current study. DANLIFE contains information on childhood adversity for all children born in Denmark between January 1st 1980 and December 31st 2015. In this study, a sample was used consisting of children born 1980–2001, who could be followed their entire childhood until 15 years of age (*n* = 1,283,955). The DCR includes information on mental and physical health, such as height and weight, for all Danish men appearing at a draft board examination during the period from January 1st 2006 to October 19th 2022 (*n* = 658,943). All men who reside in Denmark are required by law to appear at the examination during the calendar year they turn 18. The examination can be postponed until age 26 upon request, but most men appear before they turn 19. We only had access to height and weight data from DCR starting from year 2006, and information on height and weight was therefore missing for a substantial part of participants born before 1988. To prevent selection bias, all DCR records of men born before 1988 (*n* = 34,054) were excluded. The current study sample consists of a total of 359,783 men born in 1988 or later, who are both in the DANLIFE cohort and have information on height and weight assessed at a draft board examination.

### Childhood adversity

Childhood adversity trajectories were derived from an earlier study using DANLIFE data [6]. In that study, group-based multi-trajectory modeling (using package TRAJ for Stata) was applied to the yearly count of adversities from 0–15 years across the following three dimensions: material deprivation (i.e., poverty and parental long-term unemployment), loss or threat of loss (i.e., parental or sibling somatic illness or death), family dynamics (i.e., foster care placement, maternal separation, parental alcohol or drug abuse, parental or sibling psychiatric illness). Zero-inflated Poisson regressions with a cubic trajectory function were used to model childhood adversities, resulting in the identification of five groups of adversity trajectories: (1) *Low adversity* is characterized by yearly adversity counts close to zero within all three assessed dimensions. (2) *Early life material deprivation* is characterized by a high rate of material deprivation during the first years of life followed by a decreasing rate of yearly counts on this dimension. The yearly count of adversities across the other two dimensions was close to zero for this group. (3) *Persistent material deprivation* is characterized by a high rate of material deprivation throughout childhood combined with yearly counts close to zero on the other two dimensions. (4) *Loss or threat of loss* is characterized by a moderate to high and increasing rate of loss or threat of loss across childhood, including death or somatic illness in the family, combined with relatively low rates in the two other dimensions. (5) *High adversity* is characterized by a high and increasing annual rate of adversities in all three dimensions. For a graphical visualization of the trajectories, please see Rod et al. [6]. The strong association of these five trajectory groups with later mortality and morbidity attests to their adequacy and validity [6, 10, 29, 30]. All individuals in the current study were allocated to the adversity trajectory they were most likely to belong to. The childhood adversity groups were thus included as exhaustive and exclusive categorical variable in all analyses.

### Body mass index in young adulthood

BMI is calculated using height and weight measured at the draft board examination. The examinations are carried out by medical staff using standardized methods for measurement of height and weight. BMI was used as a categorical variable applying the weight categories defined by the World Health Organization (WHO): Underweight BMI < 18.5; Normal weight BMI 18.5–24.9; Overweight BMI 25.0–29.9; Obesity BMI ≥ 30.0.

### Covariates

Several potential confounders of the association between childhood adversity and weight status in young adulthood were considered based on previous research. Information on birth year, age at draft board examination, parental country of origin, maternal age at birth, parental cardiometabolic disease, preterm birth, size for gestational age, and parental education was extracted from Danish nationwide registries. Birth year refers to the calendar year the child was born. Age at draft board examination is a continuous variable and refers to the age the individual had when appearing at the draft board examination. Parental country of origin is a binary variable classifying parents as either Western (if one or both parents had a European, North American, Australian, or New Zealand nationality) or non-Western (if both parents had another nationality). Maternal age was applied as a categorical variable classifying mother’s age at birth as younger (<20 years), average (20–30 years), or older (>30 years). Parental cardiometabolic disease in the 3 years before child birth was identified in either the Danish National Patient register or the Danish Register of Causes of Death as the presence of any of the following diagnoses: ischemic heart disease (ICD8: 410–414/ICD10: I20.0, I20.1, I21–159, I25), cerebrovascular disease (ICD8: 430–438/ICD10: I60–I69), congestive heart failure (ICD8: 427.09–427.11, 427.19, 160 428.99, 782.49/ICD10: I50, I11.0, I13.0, I13.2), peripheral vascular disease (ICD8: 440–445/ICD10: I70–I74, I77), type 1 diabetes (ICD8: 249/ICD10: E10), or type 2 diabetes (ICD8: 250/ICD10: E11). Preterm birth was a binary variable classifying the children as “preterm” if they were born prior to gestational week 37 and as “not preterm” if they were born in gestational week 37 or later. Size for gestational age was used as a categorical variable depicting small (<10th), average (≥10th–≤90th), and large (>90th) gestational size at birth according to percentiles of age- and sex-specific intrauterine growth reference curves. Parental education refers to the household’s highest educational level at the time of birth and was used as a categorical variable classifying parental education as either low ( < 10 years) corresponding to elementary school, medium (10–12 years) corresponding to high-school, or high (>12 years) corresponding to a higher degree.

### Statistical analyses

We used descriptive statistics to examine participant characteristics. Associations of the childhood adversity trajectories with the categorical weight status variable were analyzed using multinomial logistic regression models, with low adversity as the reference group [31]. Model 1 (the main model) included age at draft board examination, birth year, parental origin, and maternal age where the relationships between the link function and the covariates birth year and age at draft board examination were modeled with flexible splines. Parental cardiometabolic disease, size for gestational age, and preterm birth may have been influenced by family adversity before birth of the child, whereas parental education is closely related to childhood adversity, especially the dimension of material deprivation. Thus, these variables were not included in the main analysis, but were instead presented in three supplementary models: Supplementary Model 1 included parental cardiometabolic disease in addition to variables in Model 1. Supplementary Model 2 additionally included size for gestational age and preterm birth. Finally, Supplementary Model 3 included parental education in addition to the covariates in Supplementary Model 1.

## Results

The majority (58.2%) of men belonged to the *low adversity* group, 20.1% were in the *early material deprivation* group, 9.7% in the *persistent material deprivation* group, 9.4% in the *loss or threat of loss* group, and 2.6% of the men belonged to the *high adversity* group.

Table 1 presents participant characteristics according to childhood adversity trajectory. The largest differences between the adversity groups were observed for maternal age and parental education. The *low adversity* group had a lower percentage of mothers in the younger category and a higher percentage of parents with a higher education than the other adversity groups. Table 2 presents the frequency of underweight, normal weight, overweight, and obesity across the five adversity groups. Group differences were observed across all weight groups with the lowest percentage of underweight and obesity observed in the *low adversity* group, whereas the highest percentage of underweight and obesity was observed in the *high adversity* group.

**Table 1:** Participant characteristics in the total sample and across adversity trajectory group.

	Low adversity (*n* = 209,218)	Early life material deprivation (*n* = 72,419)	Persistent material deprivation (*n* = 35,074)	Loss or threat of loss (*n* = 33,781)	High adversity (*n* = 9291)	Total (*n* = 359,783)
Birth year, median (IQR)	1995 (1991, 1998)	1993 (1990, 1996)	1993 (1990, 1997)	1995 (1992, 1998)	1993 (1990, 1996)	1994 (1991, 1997)
Age, draft board examination, median (IQR)	18.9 (18.7, 19.5)	18.9 (18.7, 19.7)	18.9 (18.7, 19.6)	18.9 (18.7, 19.5)	19 (18.7, 19.6)	18.9 (18.7, 19.5)
Parental origin, *n*(%)						
Non-western	2381 (1.1)	3192 (4.4)	4393 (12.5)	1287 (3.8)	205 (2.2)	11,458 (3.2)
Western	206,837 (98.9)	69,227 (95.6)	30,681 (87.5)	32,494 (96.2)	9086 (97.8)	348,325 (96.8)
Maternal age, *n*(%)						
Young (<20 years)	1364 (0.7)	2070 (2.9)	1680 (4.8)	1013 (3.0)	773 (8.3)	6900 (1.9)
Average (20–30 years)	130,170 (62.2)	51,087 (70.5)	23,735 (67.7)	20,694 (61.3)	6203 (66.8)	231,889 (64.5)
Old (>30 years)	77,684 (37.1)	19,262 (26.6)	9659 (27.5)	12,074 (35.7)	2315 (24.9)	120,994 (33.6)
Parental cardiometabolic disease, *n*(%)						
No	207,871 (99.4)	71,921 (99.3)	34,775 (99.1)	32,792 (97.1)	9162 (98.6)	356,521 (99.1)
Yes	1347 (0.6)	498 (0.7)	299 (0.9)	989 (2.9)	129 (1.4)	3262 (0.9)
Size for gestational age, *n*(%)						
Small	20,714 (10.1)	8519 (12.0)	4479 (13.1)	4354 (13.2)	1818 (20.1)	39,884 (11.3)
Average	157,469 (76.9)	54,086 (76.5)	25,950 (75.9)	24,740 (75.0)	6502 (72.0)	268,747 (76.4)
Large	26,477 (12.9)	8129 (11.5)	3761 (11.0)	3877 (11.8)	712 (7.9)	42,956 (12.2)
Missing	4558	1685	884	810	259	8196
Preterm birth, *n*(%)						
No	194,660 (94.7)	67,089 (94.5)	32,279 (94.0)	30,685 (92.7)	8278 (91.4)	332,991 (94.3)
Yes	10,819 (5.3)	3894 (5.5)	2049 (6.0)	2411 (7.3)	781 (8.6)	19,954 (5.7)
Missing	3739	1436	746	685	232	6838
Parental education, *n*(%)						
Low (<10 years)	13,155 (6.3)	13,562 (18.8)	9132 (26.2)	6107 (18.1)	4672 (50.6)	46,628 (13.0)
Average (10–12 years)	101,567 (48.6)	40,472 (56.1)	17,928 (51.4)	17,331 (51.4)	3507 (38.0)	180,805 (50.4)
High (>12 years)	94,357 (45.1)	18,170 (25.2)	7808 (22.4)	10,279 (30.5)	1048 (11.4)	131,662 (36.7)
Missing	139	215	206	64	64	688

*IQR* inter quartile range.

**Table 2:** Prevalence of weight categories in the total sample and across adversity trajectory groups.

	Low adversity (*n* = 209,218)	Early life material deprivation (*n* = 72,419)	Persistent material deprivation (*n* = 35,074)	Loss or threat of loss (*n* = 33,781)	High adversity (*n* = 9291)	Total (*n* = 359,783)
Underweight, *n*(%)	10,423 (5.0)	3901 (5.4)	1977 (5.6)	1879 (5.6)	587 (6.3)	18,767 (5.2)
Normal weight, *n*(%)	143,080 (68.4)	46,475 (64.2)	21,975 (62.7)	21,806 (64.6)	5918 (63.7)	239,254 (66.5)
Overweight, *n*(%)	42,125 (20.1)	15,732 (21.7)	7808 (22.3)	7134 (21.1)	1891 (20.4)	74,690 (20.8)
Obesity, *n*(%)	13,590 (6.5)	6311 (8.7)	3314 (9.4)	2962 (8.8)	895 (9.6)	27,072 (7.5)

**Fig. 1:**
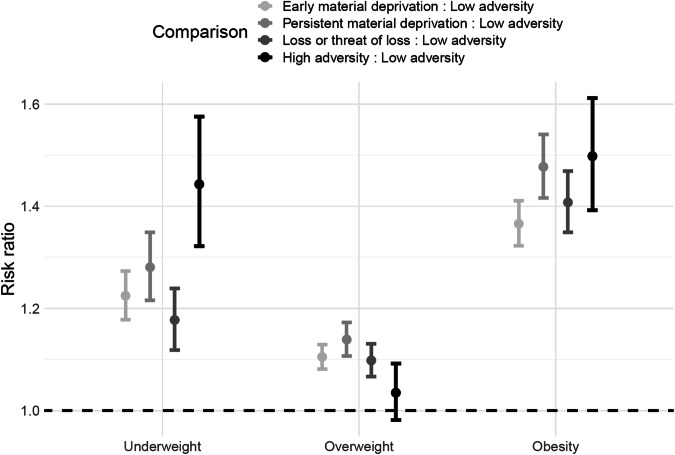
Associations of childhood adversity trajectory groups with underweight, overweight and obesity among 359,783 men from the DANLIFE cohort. The figure presents risk ratios (RR) with 95% confidence intervals (CI) for underweight, overweight, and obesity for each adversity group compared with the low adversity group. The RRs are adjusted according to Model 1: birth year, age at draft board examination, parental origin, and maternal age.

Results of the multinomial regression models investigating associations of adversity trajectory groups and weight status category are presented in Fig. 1 and Supplementary Fig. S1a–c. The plots present risk ratios (RR) for having underweight, overweight, and obesity compared with normal weight. RR estimates for all analyses are presented in Supplementary Table 1. Overall, results show that men in the adversity groups have higher risks of underweight, overweight, or obesity, compared with the *low adversity* group. In the main model, adjusted for birth year, age at draft board examination, parental origin, and maternal age (Fig. 1), the strongest associations with underweight (RR 1.44; 95% CI: 1.32, 1.58) and obesity (1.50; 1.39, 1.61) were found for the *high* vs. *low adversity* group. The highest RR for overweight (1.14 (1.11, 1.17)) was found for the *persistent material deprivation* group compared with the *low adversity group*. These results remained similar when additionally adjusting for cardiometabolic disease (Fig. S1a), and for cardiometabolic disease, size for gestational age and preterm birth (Fig. S1b). When adjusting for parental education (Fig. S1c), the RR estimates were attenuated for all adversity groups, and particularly for the *high adversity* group. After adjustment for parental education, high adversity was associated with a slightly lower risk of overweight (0.90 (0.85, 0.95)) when compared to the *low adversity* group.

## Discussion

Results from this large prospective population-based study showed that young men with a history of childhood adversity during the first 15 years of life had a higher risk of underweight, overweight, and obesity compared with men who experienced low childhood adversity. For instance, men in the *high adversity* group had a 44% and 50% higher risk of underweight and obesity respectively, than men in the *low adversity* group. These results were not explained by confounding from risk factors such as parental origin, maternal age, parental cardiometabolic disease, size for gestational age, or preterm birth. However, results were attenuated when adjusting for parental education.

A growing body of research has investigated associations of adverse childhood experiences and adult obesity, and the findings are generally in line with those of the present study. A systematic review of the literature (*n* = 253,719) combining cross-sectional, case-control, and cohort studies found an odds ratio for adult overweight or obesity of 1.39 (CI = 1.13, 1.71) among individuals who had been exposed to childhood adversity when compared with individuals who had not [5]. A more recent review of ten population-based cross-sectional studies (*n* = 118,691) found a 46% higher odds of obesity (OR(CI)1.46 (1.28, 1.64)) in adulthood following exposure to multiple adverse childhood experiences [11]. Few studies have investigated longitudinal associations between childhood adversities and adult obesity specifically. A cross-sectional study of 26,615 mid to late life individuals who were asked to recall exposure on childhood adversities identified a dose-repose relationship between number of adversities and risk of obesity [9]. Adults who reported four to eight adversities had 54% higher risk of obesity (1.54 (1.28, 1.75)) than adults who did not report adversities in childhood. A meta-analysis of associations between accumulation of adversity or trauma in childhood and overweight during childhood and adolescence [32] found a pooled OR of 1.12 (1.01, 1.25), which is substantially lower than the OR reported in studies of adult obesity. This may indicate that it takes several years for childhood adversities to manifest their effects on obesity in adulthood [33]. Our study contributes to these previous findings by using prospectively collected information on childhood adversity from a large population-based sample with measured BMI in young adulthood, which is a critical window for later weight developments.

It is highly plausible that exposure to childhood adversity combined with genetic predisposition contributes to obesity development both within and across generations [34]. A systematic review of population-based studies found that social disruption, health behavior, and chronic stress responses were the most likely explanations for the association between childhood adversity and adult obesity [11]. Several other mechanisms may contribute to explaining the observed association. A recent theory suggests that childhood adversity triggers an evolutionary developed mechanism, which originally had the function of storing fat when the prospect of having social support and therefore food was uncertain [20]. This may indicate that social factors such as childhood adversities have a direct effect on weight status through biological pathways. This notion is supported by findings linking early stressful life conditions and social factors, such as poverty, to increased HPA axis reactivity that may lead to increased fat deposits [11, 35]. Another possible explanation is that childhood adversity has indirect effects on weight status in adulthood by inducing other factors with presumed direct influence on obesity development. Individuals who have been exposed to childhood adversity are more likely to engage in unhealthy behaviors such as eating a poor quality diet [17] and limited physical activity [36]. Also, early adverse experiences have consistently been linked to negative psychological outcomes such as depression [37, 38], which in turn are known to influence an individuals’ eating patterns and weight status [39].

In the current study, childhood adversity was also associated with underweight. Specifically, men in the high adversity group had a higher risk of underweight than men in the low adversity group. While previous results are overall inconsistent [22, 33], the association between childhood adversities and underweight has been found in previous studies [40–42]. Childhood adversities may lead to the development of underweight through psychological and behavioral factors, such as poor mental health [43, 44] maladaptive coping and self-harm behaviors including drug abuse and restricted or disordered eating [45, 46]. Also, a low BMI in young adulthood may be indicative of chronic physical disease, which has also been associated with childhood adversities.

There is a conceptual overlap between parental education and childhood adversity, particularly concerning the material deprivation dimension within trajectories, which include indicators such as poverty and prolonged parental unemployment. It is therefore interesting to see that associations of childhood adversities with underweight persisted even when adjusting for parental education, while they as expected attenuated for obesity (Supplementary Model 3). This indicates that the underlying mechanisms linking childhood adversities with body weight may differ across weight categories. The association of childhood adversity with overweight (BMI ≥ 25) was modest indicating that childhood adversity may be an important predictor of the more extreme weight categories (i.e., underweight and obesity) rather than the weight categories within the broader normal range.

A major strength of the present study is the prospective population-based design minimizing recall bias and selective inclusion. Additional strengths include the large sample, the nationwide register-based information on yearly incidence of different childhood adversities and their accumulation across childhood with proven associations with later morbidity and mortality [6, 10]. Finally, the study included extensive information on potential early life confounding factors.

Some limitations of this study are that we did not have direct measurements of childhood adversities, such as family violence, sexual abuse, or neglect, nor measures on social dynamics in schools such as bullying, which have been related to various health outcomes [47]. Neither do we have information on manifested effects of the adversities on the individual children, which was the key exposure in a previous study, showing a much stronger relation of parental neglect in childhood with later obesity in young adulthood [48]. Likewise, we did not have information on parental BMI, which is a well-established genetic and shared familial environmental predictor of the child’s BMI, and it may also affect the conditions under which the child grow up [49, 50]. This is especially relevant for the *loss and threat of loss* group, which is often associated with parental somatic disease. We did, however, adjust for parental cardiometabolic disease as a crude indicator of parental BMI, which only had very minor impact on the results. Furthermore, we did not have information on other weight status indicators than BMI. This is a limitation as the accuracy of BMI on the individual level may be questioned.

Finally, the study sample included only men, and some previous studies have indicated sex differences in the association between childhood adversities and obesity with a stronger association among women than men [51, 52]. Thus, this suggests that the associations in our study might be stronger had we had the possibility to study them in young women. Overall, the current study provides solid evidence of an association between adversity trajectories in childhood and young adult weight status. Future research should focus on investigating the underlying mechanisms of this association, for instance, whether the risk of underweight and obesity is driven by different types of adversities. Also, there is a need for large population-based studies investigating the effect of adversity trajectories in childhood on weight status throughout adulthood.

## Conclusion

Findings from this large population-based study showed that men who had encountered childhood adversity within the first 15 years of their life faced a higher risk of underweight, overweight, and obesity in young adulthood when compared with those with low adversity. These findings emphasize the importance of early life experiences in shaping healthy weight trajectories across the lifespan. Young adulthood represents a critical phase in weight development, carrying potential implications for later-life health and overall well-being. Addressing childhood adversity within social and familial contexts could serve as central intervention opportunities for public health initiatives.

### Supplementary information

Supplementary figures

## Data Availability

The data material contains personally identifiable and sensitive information. According to the Act on Processing of Personal Data, such data cannot be made publicly available. Inquiries about secure access to data under conditions stipulated by the Danish Data Protection Agency should be directed to the principal investigator of DANLIFE, NHR (nahuro@sund.ku.dk).
