# Supplementary material for: Childhood adversity trajectories and weight status in young adult men: a register-based study including 359,783 Danish men
Source: Int J Obes (Lond). 2024 May 30;48(8):1157–63. doi: 10.1038/s41366-024-01540-4 (PMC11281903; doi:10.1038/s41366-024-01540-4)
Supplement: Supplementary file 1 — Supplementary figures [file 41366_2024_1540_MOESM1_ESM.pdf]

Supplementary figure S1 (a-c): Associations of childhood adversity trajectory groups with underweight, overweight and obesity among 359 783 men from the DANLIFE cohort. The figure presents risk ratios (RR) with 95% Confidence Intervals (CI) for underweight, overweight, and obesity for each adversity group compared with the low adversity group. The RRs are adjusted according to supplementary model 1 (figure S1a): Birth year, age at draft board examination, parental origin, maternal age, and parental cardiometabolic disease; supplementary model 2 (figure S1b): Birth year, age at draft board examination, parental origin, maternal age, parental cardiometabolic disease, size for gestational age, and preterm birth; and supplementary model 3 (figure S1c): Birth year, age at draft board examination, parental origin, maternal age, parental cardiometabolic disease and parental education (1c)

Figure S1a

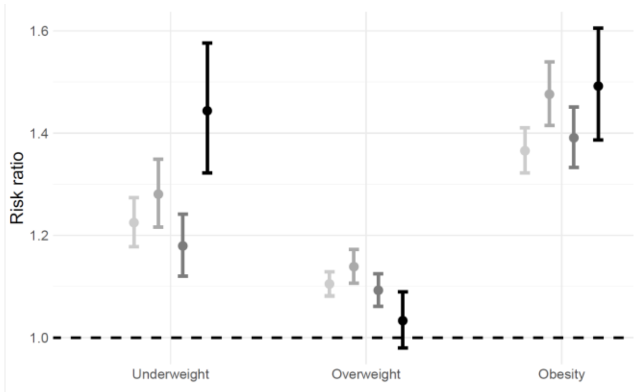

Figure S1b

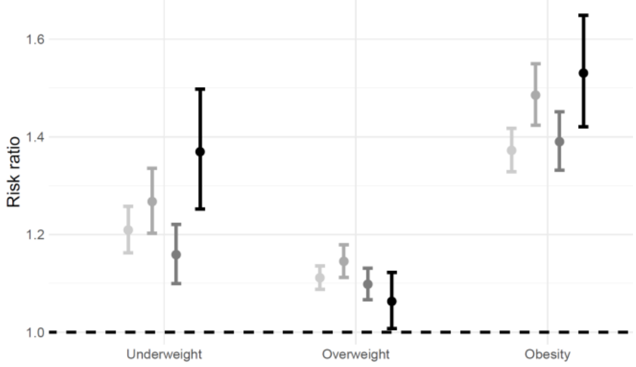

Figure S1c

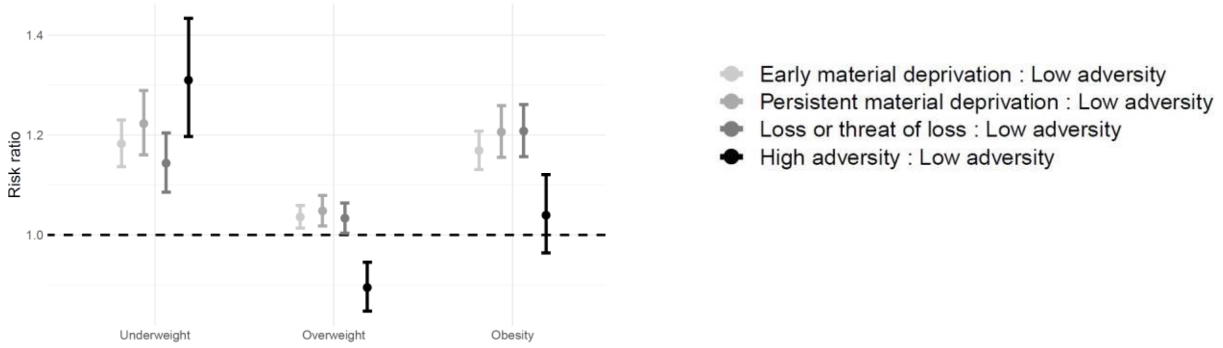

Supplementary table 1: Associations of adversity trajectory groups with weight category. The table presents risk ratios (RR) with 95% Confidence Intervals (CI) for underweight, overweight, and obesityfor each adversity group compared with the 'Low adversity' group.

	Model 1[a]		
	Underweight	Overweight	Obesity
Low adversity	1.0	1.0	1.0
Early life material deprivation	1.23 (1.18, 1.27)	1.11 (1.08, 1.13)	1.37 (1.32, 1.41)
Persistent material deprivation	1.28 (1.22, 1.35)	1.14 (1.11, 1.17)	1.48 (1.42, 1.54)
Loss or threat of loss	1.18 (1.12, 1.24)	1.10 (1.07, 1.13)	1.41 (1.35, 1.47)
High adversity	1.44 (1.32, 1.58)	1.04 (0.98, 1.09)	1.50 (1.39, 1.61)
	Supplementary model 1[b]		
Low adversity	1.0	1.0	1.0
Early life material deprivation	1.23 (1.18, 1.27)	1.11 (1.08, 1.13)	1.37 (1.32, 1.41)
Persistent material deprivation	1.28 (1.22, 1.35)	1.14 (1.11, 1.17)	1.48 (1.42, 1.54)
Loss or threat of loss	1.18 (1.12, 1.24)	1.10 (1.06, 1.13)	1.39 (1.33, 1.45)
High adversity	1.44 (1.32, 1.58)	1.03 (0.98, 1.09)	1.49 (1.39, 1.61)
	Supplementary model 2[c]		
Low adversity	1.0	1.0	1.0
Early life material deprivation	1.21 (1.16, 1.26)	1.11 (1.09, 1.14)	1.37 (1.33, 1.42)
Persistent material deprivation	1.27 (1.20, 1.34)	1.15 (1.11, 1.18)	1.49 (1.42, 1.55)
Loss or threat of loss	1.16 (1.10, 1.22)	1.10 (1.07, 1.13)	1.39 (1.33, 1.45)
High adversity	1.37 (1.25, 1.50)	1.06 (1.01, 1.12)	1.53 (1.42, 1.65)
	Supplementary model 3[d]		
Low adversity	1.0	1.0	1.0
Early life material deprivation	1.18 (1.14, 1.23)	1.04 (1.01, 1.06)	1.17 (1.13, 1.21)
Persistent material deprivation	1.22 (1.16, 1.29)	1.05 (1.02, 1.08)	1.21 (1.16, 1.26)
Loss or threat of loss	1.14 (1.09, 1.21)	1.03 (1.00, 1.06)	1.21 (1.16, 1.26)
High adversity	1.31 (1.20, 1.43)	0.90 (0.85, 0.95)	1.04 (0.96, 1.12)

[a]adjusted for birth year, age at draft board examination, parental origin, and maternal age
[b]adjusted for birth year, age at draft board examination, parental origin, maternal age, and parental cardiometabolic disease
[c] adjusted for birth year, age at draft board examination, parental origin, maternal age, parental cardiometabolic disease, size forgestational age, and preterm birth
[d] adjusted for birth year, age at draft board examination, parental origin, maternal age, parental cardiometabolic disease, and parentaleducation
